# Urological Cancer Panorama in the Second Year of the COVID-19 Pandemic

**DOI:** 10.3390/cancers14030493

**Published:** 2022-01-19

**Authors:** Estibaliz López-Fernández, Javier C. Angulo, José I. López, Claudia Manini

**Affiliations:** 1 FISABIO Foundation, 46020 Valencia, Spain; estibaliz.lopez@universidadeuropea.es; 2 Faculty of Health Sciences, European University of Valencia, 46023 Valencia, Spain; 3 Clinical Department, Faculty of Medical Sciences, European University of Madrid, 28005 Madrid, Spain; javier.angulo@universidadeuropea.es; 4 Department of Urology, University Hospital of Getafe, 28907 Madrid, Spain; 5 Department of Pathology, Cruces University Hospital, 48903 Barakaldo, Spain; 6 Biocruces-Bizkaia Health Research Institute, 48903 Barakaldo, Spain; 7 Department of Pathology, San Giovanni Bosco Hospital, 10154 Turin, Italy; 8 Department of Public and Pediatric Health Sciences, University of Turin, 10124 Turin, Italy

A total of 22 contributions conforms this Special Issue that covers a wide spectrum of contemporary issues in urological cancer, a group of neoplasms with high incidence, prevalence, and mortality rates, especially in the male population of Western countries [1]. Renal cancer, with five contributions (three articles, one communication, and one perspective), prostate cancer and allied conditions, with ten (eight articles, one communication, and one review), and urinary bladder, with seven (five articles and two reviews), provide a comprehensive panorama of what has occurred in Urologic Oncology during 2021, the second year of the COVID-19 pandemic. Most papers in this collection deal with different aspects of cancer therapy, but diagnostic and prognostic subjects, migration-related topics, reviews, and preneoplastic lesions are also considered. Overall, this translational compilation of viewpoints around urological cancer will enrich multidisciplinary interactions for patient benefit.

## 1. Renal Cancer

Three papers analyze three different approaches to face against renal cancer, that is, surgery, radiotherapy, and immune checkpoint inhibition, thus reflecting the broad diversity of therapeutic possibilities available in this complex disease. In a context of different treatments, a precise definition of strict criteria for a rationale patient selection would be advisable.

Gonzalez et al. [2] analyze the post-surgical complications and survival benefit of radical surgery in a series of 18 locally advanced clear cell renal cell carcinomas (CCRCC) invading the inferior vena cava, pancreas, duodenum, and liver, establishing the technical feasibility of a radical resection that includes complex procedures derived from transplant surgery. A deep surgical experience seems, however, mandatory for a successful implementation of this therapeutic option. The usefulness of the stereotactic body radiotherapy as an alternative treatment to surgery has been explored by Grelier et al. [3]. Twenty-three patients have been treated with this option during a 4-year period. All the cases had a biopsy for pathological confirmation or renal cell carcinoma (RCC) prior to the procedure. All were organ-confined tumors (T1/2) and, as expected, CCRCC was the most frequently found neoplasm (73.9%). The dose administered oscillated between 24 Gy and 35 Gy and fractions from 3 and 5. The obtained results indicate that this technique could be seriously considered as a promising therapeutic alternative for patients with poor physical health. However, further studies are necessary to support the exact role of this treatment in RCC. PD-1/PD-L1 axis blockade drugs, alone or in combination with other drugs, are being introduced in the therapeutic armamentarium in several neoplasms, advanced CCRCC included. However, its benefit to patients is far from being generalized and some controversies still exist in patient selection [4]. Shiuan et al. [5] have analyzed the patient characteristics, clinical correlates, and molecular parameters (multiplexed immunofluorescence, whole exome sequencing, T-cell receptor sequencing, RNA sequencing) in a series of RCC (clear cell, papillary, sarcomatoid, chromophobe, and undifferentiated) treated with nivolumab (84%) and atezolizumab (16%) in first (8.5%), second (29.8%), third (34%) and fourth (27.7%) lines. The authors conclude that PD-L1 immunostaining alone does not provide enough information to predict response in these patients.

Chow et al. [6] have analyzed the role of OPA interacting protein 5 (OIP5) in 20 papillary renal cell carcinomas (PRCC) and 37 (CCRCC). OIP5 up-regulation has been associated with biological aggressiveness in a broad spectrum of malignant neoplasms, PRCC included. For such a purpose, the authors have constructed a 66-gene multigene panel (Overlap66), including PLK1 gene, which effectively stratifies high-risk PRCC thus allowing to treat them with PLK1 inhibitors. The authors conclude that Overlap66 analysis and PLK1 inhibitors should be added to the list of personalized therapies in PRCC.

In a perspective, Laruelle et al. [7] analyze the metastatic process of CCRCC as an example of tumor evolvability, where a sociological perspective to the problem provided by Game Theory may shed some additional light to our knowledge of cancer evolutionary mechanisms. The authors hypothesize that the development of metastases in malignant tumors respond to the necessity of a subset of tumor cells to achieve Nash equilibria and friendlier environments far away from the primary tumor.

## 2. Prostate Cancer

Three out of 10 papers in this section deal with advanced prostate cancer. Fourquet et al. [8] evaluate the role of [68Ga]Ga-PSMA-11 PET/CT in predicting therapy efficacy, diagnostic usefulness, and patient management in 294 patients with biochemical recurrence. The authors conclude that this technique shows a high performance in locating prostate cancer recurrence sites. Additionally, [68Ga]Ga-PSMA-11 PET/CT impacts therapeutic management in two out of three patients studied. The use of PSMA radioligands with PET/CT should be considered as a first line imaging in patients with biochemical recurrence. Patient outcomes and hormonal therapy patterns have been retrospectively studied in a large observational real-world database of patients with metastatic prostate cancer in the USA by Swami et al. [9]. The authors conclude that novel hormonal therapies and doxetacel were underutilized in the series analyzed. In the third contribution within this subheading, Rasul et al. [10] analyze the response and toxicity of three cycles of 177Lu-PSMA in patients with castration-resistant prostate cancers. The authors conclude on their study that an intensive PSMA-radioligand therapy is well tolerated by patients with metastatic castration-resistant prostate cancers and is associated with promising overall survivals.

Four contributions analyze different aspects of the diagnostic process in prostate cancer. For example, Fulco et al. [11] evaluate the diagnostic accuracy of multiparametric magnetic resonance imaging-ultrasound fusion trans-perineal prostate biopsy. This single center retrospective study of 272 patients shows that a total of 16.7% of clinically significant tumors would have been undetected with standard biopsy methods. The authors conclude that the combined targeted and standard biopsy methodology is advisable to minimize the risk of missing clinically significant prostate cancer. Myint et al. [12] analyze the immunohistochemical expression of glutaminase in a series of 154 cancer and 41 benign prostate samples. RNA-Seq data of 246 prostate cancer samples are also obtained from The Cancer Genome Atlas. The authors conclude that although glutaminase expression is higher in prostate cancer than in benign prostate tissue this difference does not seem to be statistically significant. Connell et al. [13] develop a multivariable risk model termed ExoGrail for the non-invasive detection of prostate cancer prior to biopsy. They have observed that this model is able to reduce the number of unnecessary biopsies by 35% when compared with current standards of care. Selvaggio et al. [14] use a fluorescent confocal microscope to intraoperatively analyze the ablation margins in 10 patients to improve partial prostate gland cryoablation outcomes. They conclude that this technique is feasible and reliable to reduce disease recurrence and functional complications of focal therapy in prostate cancer.

Neupane et al. [15] have developed a full prognostic index for predicting the survival of localized prostate cancer up to 15 to 20 years using a multivariable complementary log-log regression model. Age at diagnosis, trial arm, PSA at diagnosis, European Association Urology (EAU) risk group, treatment modality, mode of detection, and biochemical recurrence are taken into account in the study but not co-morbidity as it has not got a great impact on prostate cancer-specific survival. A simplified risk score tool has also been developed for early diagnosis and to predict survival at 10 years considering the three parameters commonly used in daily clinical practice (age, PSA, and EAU risk group). Both the full and simplified prognostic index have shown a superior performance than the EAU risk group, being the latest also more accurate in risk estimation than D’Amico risk classification and the cancer of prostate risk assessment (CAPRA) risk score. The authors conclude that further validations of both prognostic indexes are needed.

Kimura et al. [16] review the global trends of latent prostate cancer in Western and Asian countries considering variables such as step-sectioning versus random and/or single-sectioning, thickness, age, and race. The increased prevalence observed over time in Asia, as compared to the stable numbers among Western countries, could be explained due to PSA screening strategies and changes to Westernized lifestyles. The authors conclude that it is mandatory to agree in the diagnostic methods and molecular analyses to obtain homogeneous data that could even reconsider the nowadays definition of latent prostate cancer.

De Godoy Fernandes et al. [17] analyze the proliferative inflammatory atrophy in canine prostatic samples as a preneoplastic lesion with potential progression to prostate cancer. Proliferative atrophy shows a high proliferative rate in concordance with the overexpression observed in Ki67, CK5, high molecular weight cytokeratins, and p63. On the other hand, p53 and MDM2 are not deregulated as they are in advanced stages of prostatic carcinogenesis. Androgen receptor and PTEN are both downregulated activating the anti-apoptotic pathway. The authors conclude that high proliferative indexes and low levels of androgen receptor and PTEN are useful biomarkers to predict potential preneoplastic lesions even though more studies should be conducted.

## 3. Bladder Cancer

Two out of seven papers in this section are reviews. Rebuzzi et al. [18] evaluate the prognostic and predictive factors of advanced urothelial carcinoma treated with immune checkpoints inhibitors (ICIs), with the intention to identify biomarkers to select the patients at higher chances of responding to ICI treatment that may merit further prospective investigation. Their main conclusions are that current evidence on the predictive and prognostic value of PD-L1 expression is limited by the different assays used for each anti-PD1 or anti-PD-L1 agent in the clinical trials evaluated, and thereof their clinical value remains inconclusive. On the other hand, the authors position towards the value of sequencing of the tumor fraction of the cell-free DNA (ctDNA) is an interesting method to detect residual disease and anticipate disease relapse after treatment using different biomarkers that include FGFR3, XPD, HER2, and TMB. What is more, the serial monitoring of ctDNA as a tumor tissue surrogate could be of value to stratify metastatic urothelial carcinoma and could act as a treatment response marker [18,19].

Silina et al. [20] reviews bladder sparing strategies with the use of radiotherapy and chemotherapy combined, a very interesting therapeutic approach for invasive bladder cancer, that is emerging also as a therapeutic possibility in the context of medical system collapse for scheduled surgeries suffered by the COVID-19 health crisis. Based on the issue that strategies to radio-sensitize tumors and spare normal bladder tissue to improve treatment safety, a systematic search and literature review is performed. The conclusion is that a long way remains ahead before experimental research with cell lines and animal models regarding the combination of radiation with different agents can be optimized.

Five additional articles present original investigations regarding invasive bladder cancer. Miyake et al. [21] present a retrospective study of a very large series of patients with T1 high-grade urothelial carcinoma and evaluate the prognostic role of divergent differentiation and variant morphologies when Bacillus Calmette-Guérin (BCG) endovesical treatment is used. This topic presents the clinical implications of urothelial cancer heterogeneity and is of particular interest in this era of BCG shortage, where new treat modalities such as chemo-hyperthermia to optimize adjuvant treatment after transurethral resection are being investigated [22,23] or even early cystectomy [24] are controversial. With the limitations of the study design using inverse probability of treatment weighting analysis, the authors found that not only recurrence-free or progression-free rates are affected, but also cancer-specific mortality was affected by variant morphologies (nested, microcystic, micropapillary, lympho-epithelioma-like, plasmacytoid, giant cell, sarcomatoid, lipid-rich and clear cell variants), but not for urothelial cancer with divergent (squamous, glandular and trophoblastic) differentiation. This analysis is very interesting to optimize BCG therapy and promote a radical treatment once variant morphologies are identified.

The remining four original articles on invasive bladder cancer in the Issue deal the eternal dilemma to find the optimal prognostic marker in patients with invasive bladder cancer treated with radical cystectomy. It is still surprising that despite decades of intensive research the optimal tissue markers for urothelial cancer remains undetermined. Recent developments regarding basal/luminal phenotype markers are consolidating to predict disease prognosis, but other new immunohistochemical evaluations such as fibroblast activating protein (FAP) or pro-renin receptor (PRR) are also newly discovered [25,26].

Two very interesting novel pathways and their prognostic implications are investigated in the Special Issue. Koguchi et al. 2021 [27] evaluate the expression of AHNAK2 (AHNAK Nucleoprotein 2), a protein coding gene, that has been identified as a possible tumor marker and therapeutic target in different malignancies including clear cell renal cell carcinoma, papillary thyroid carcinoma, lung and pancreatic duct adenocarcinoma, and others. Their study of 120 patients with bladder cancer undergoing radical cystectomy finds an association between high AHNAK2 expression and classical aggressive pathologic findings, but also as an independent prognostic marker for recurrence-free survival and disease-specific mortality in this set of patients [27]. Chen et al. [28] investigate S1PR1 expression, a G protein-coupled receptor in vascular endothelial and immune cells. Their experimental cell-line and human tissue approach using transurethral resection specimens, and also the search in gene expression database collection NCBI-GEO, has evidenced that high S1PR1 expression in neoplasia inversely correlates with cell motility; thus, targeting *S1PR1* may result in the enhanced migration of bladder cancer cells. The implications of this finding merit further research to inhibit the progression of metastases.

The development of bladder cancer diagnostic markers is an unmet need in current Urologic Oncology. Shimura et al. [29] use a micro-dot blot array to evaluate EPPK1 (epiplakin) expression in patients with bladder neoplasia and controls. Their results are very stimulating because this cytoskeletal linker protein, that connects to intermediate filaments and controls their reorganization, is a promising molecule to act as diagnostic biomarkers for patients with urothelial carcinoma of the bladder. As revealed, the area under the curve is 78%. Immunohistochemical evaluation in a series of 127 patients with a bladder tumor treated with radical cystectomy did not confirm the role of this marker with cancer prognosis. Of course, validation and large-scale studies are needed to confirm these very promising results.

Finally, Sugino et al. [30] have evaluated another impressive prognosticator for bladder cancer, based on imaging studies alone. The preoperative non-contrast CT-scan evaluation axial image at the third lumbar vertebral level of psoas muscle Hounsfield unit before radical cystectomy correlates with the clinical prognosis. This factor, assessed in 177 consecutive surgically treated patients, behaves as a major predictor of overall survival, together with patient age, patient sex, and clinical staging of the disease. Of course, this is not a specific tumor marker as it probably reflects patient frailty before surgery. Tentatively, this prognostic factor can be used to predict overall survival, regardless of the condition of bladder cancer itself. Therefore, its use in Urologic Oncology can be deemed spurious. However, based on the fact that imaging modalities for preoperative assessment of patients undergoing cystectomy are universally used, its potential to be incorporated in preoperative assessment tools merits validation.

## 4. Conclusions

Patients with urological cancer are at a higher risk to be more severely affected by the infection than the general population due to their inherent immunosuppression status either related directly to the disease or secondarily induced by chemotherapy, radiotherapy, immune checkpoint inhibition, or other oncologic treatments. The global shut-down provoked by the SARS-CoV-2 pandemic in 2020 forced the urologists to reorganize their routine activity prioritizing the surgical activity and deferring non-urgent pathologies in the wake of the limited operating room capacities and hospital beds occupied by, or reserved for, the avalanche of patients with severe respiratory symptoms needing urgent medical care. Unfortunately, the high mutational capacity of the virus makes the resolution of this global problem a pending issue.

## References

[1] Siegel R.L., Miller K.D., Fuchs H.E., Jemal A. (2021). Cancer statistics, 2021. CA Cancer J. Clin..

[2] González J., Gaynor J., Ciancio G. (2021). Renal cell carcinoma with or without tumor thrombus invading the liver, pancreas and duodenum. Cancers.

[3] Grelier L., Baboudjian M., Gondran-Tellier B., Couderc A., McManus R., Deville J., Carballeira A., Delonca R., Delaporte V., Padovani L. (2021). Stereotactic body radiotherapy for frail patients with primary renal cell carcinoma: Preliminary results after 4 years of experience. Cancers.

[4] Nunes-Xavier C.E., Angulo J.C., Pulido R., López J.I. (2019). A critical insight into the clinical translation of PD-1/PD-L1 blockade therapy in clear cell renal cell carcinoma. Curr. Urol. Rep..

[5] Shiuan E., Reddy A., Dudzinski S., Lim A., Sugiura A., Hongo R., Young K., Liu X., Smith C., O’Neal J. (2021). Clinical features and multiplatform molecular analysis assist in understanding patient response to anti-PD-1/PD-L1 in renal cell carcinoma. Cancers.

[6] Chow M., Gu Y., He L., Lin X., Dong Y., Mei W., Kapoor A., Tang D. (2021). Prognostic and therapeutic potential of the OIP5 network in papillary renal cell carcinoma. Cancers.

[7] Laruelle A., Manini C., Iñarra E., López J. (2021). Metastasis, an example of evolvability. Cancers.

[8] Fourquet A., Lahmi L., Rusu T., Belkacemi Y., Créhange G., de la Taille A., Fournier G., Cussenot O., Gauthé M. (2021). Restaging the biochemical recurrence of prostate cancer with [68Ga]Ga-PSMA-11 PET/CT: Diagnostic performance and impact on patient disease management. Cancers.

[9] Swami U., Sinnott J., Haaland B., Sayegh N., McFarland T., Tripathi N., Maughan B., Rathi N., Sirohi D., Nussenzveig R. (2021). Treatment pattern and outcomes with systemic therapy in men with metastatic prostate cancer in the real-world patients in the United States. Cancers.

[10] Rasul S., Wollenweber T., Zisser L., Kretschmer-Chott E., Grubmüller B., Kramer G., Shariat S., Eidherr H., Mitterhauser M., Vraka C. (2021). Response and toxicity to the second course of 3 cycles of 177Lu-PSMA therapy every 4 weeks in patients with metastatic castration-resistant prostate cancer. Cancers.

[11] Fulco A., Chiaradia F., Ascalone L., Andracchio V., Greco A., Cappa M., Scarcia M., Ludovico G., Pagliarulo V., Palmieri C. (2021). Multiparametric magnetic resonance imaging-ultrasound fusion transperineal prostate biopsy: Diagnostic accuracy from a single center retrospective study. Cancers.

[12] Myint Z., Sun R., Hensley P., James A., Wang P., Strup S., McDonald R., Yan D., Clair W.H.S., Allison D. (2021). Evaluation of glutaminase expression in prostate adenocarcinoma and correlation with clinicopathologic parameters. Cancers.

[13] Connell S., Mills R., Pandha H., Morgan R., Cooper C., Clark J., Brewer D. (2021). Integration of urinary EN2 protein & cell-free RNA data in the development of a multivariable risk model for the detection of prostate cancer prior to biopsy. Cancers.

[14] Selvaggio O., Falagario U., Bruno S., Recchia M., Sighinolfi M., Sanguedolce F., Milillo P., Macarini L., Rastinehad A., Sanchez-Salas R. (2021). Intraoperative digital analysis of ablation margins (DAAM) by fluorescent confocal microscopy to improve partial prostate gland cryoablation outcomes. Cancers.

[15] Neupane S., Nevalainen J., Raitanen J., Talala K., Kujala P., Taari K., Tammela T., Steyerberg E., Auvinen A. (2021). Prognostic index for predicting prostate cancer survival in a randomized screening trial: Development and validation. Cancers.

[16] Kimura T., Sato S., Takahashi H., Egawa S. (2021). Global trends of latent prostate cancer in autopsy studies. Cancers.

[17] de Godoy Fernandes G., Pedrina B., de Faria Lainetti P., Kobayashi P., Govoni V., Palmieri C., de Moura V., Laufer-Amorim R., Fonseca-Alves C. (2021). Morphological and molecular characterization of proliferative inflammatory atrophy in canine prostatic samples. Cancers.

[18] Rebuzzi S., Banna G., Murianni V., Damassi A., Giunta E., Fraggetta F., De Giorgi U., Cathomas R., Rescigno P., Brunelli M. (2021). Prognostic and predictive factors in advanced urothelial carcinoma treated with immune checkpoint inhibitors: A review of the current evidence. Cancers.

[19] Vandekerkhove G., Lavoie J.-M., Annala M., Murtha A.J., Sundahl N., Walz S., Sano T., Taavitsainen S., Ritch E., Fazli L. (2021). Plasma ctDNA is a tumor tissue surrogate and enables clinical-genomic stratification of metastatic bladder cancer. Nat. Commun..

[20] Silina L., Maksut F., Bernard-Pierrot I., Radvanyi F., Créhange G., Mégnin-Chanet F., Verrelle P. (2021). Review of experimental studies to improve radiotherapy response in bladder cancer: Comments and perspectives. Cancers.

[21] Miyake M., Nishimura N., Iida K., Fujii T., Nishikawa R., Teraoka S., Takenaka A., Kikuchi H., Abe T., Shinohara N. (2021). Intravesical bacillus Calmette–Guérin treatment for T1 high-grade non-muscle invasive bladder cancer with divergent differentiation or variant morphologies. Cancers.

[22] Zhao H., Chan V.W., Castellani D., Chan E.O., Ong W.L.K., Peng Q., Moschini M., Krajewski W., Pradere B., Ng C.F. (2021). Intravesical chemohyperthermia vs. Bacillus Calmette-Guerin instillation for intermediate- and high-risk non-muscle invasive bladder cancer: A systematic review and meta-analysis. Front. Surg..

[23] Plata A., Guerrero-Ramos F., Garcia C., González-Díaz A., Gonzalez-Valcárcel I., de la Morena J.M., Díaz-Goizueta F.J., Del Álamo J.F., Gonzalo V., Montero J. (2021). Long-term experience with hyperthermic chemotherapy (HIVEC) using Mitomycin-C in patients with non-muscle invasive bladder cancer in Spain. J. Clin. Med..

[24] Nishimura N., Miyake M., Iida K., Miyamoto T., Tomida R., Numakura K., Inokuchi J., Yoneyama T., Matsumura Y., Yajima S. (2021). Prognostication in Japanese patients with Bacillus Calmette-Guerin-unresponsive non-muscle-invasive bladder cancer undergoing early radical cystectomy. Int. J. Urol..

[25] Calvete-Candenas J., Larrinaga G., Errarte P., Martín A.M., Dotor A., Esquinas C., Nunes-Xavier C.E., Pulido R., López J.I., Angulo J.C. (2019). The coexpression of fibroblast activation protein (FAP) and basal-type markers (CK 5/6 and CD44) predicts prognosis in high-grade invasive urothelial carcinoma of the bladder. Hum. Pathol..

[26] Larrinaga G., Calvete-Candenas J., Solano-Iturri J.D., Martín A.M., Pueyo A., Nunes-Xavier C.E., Pulido R., Dorado J.F., López J.I., Angulo J.C. (2021). (Pro)renin Receptor is a novel independent prognostic marker in invasive urothelial carcinoma of the bladder. Cancers.

[27] Koguchi D., Matsumoto K., Shimizu Y., Kobayashi M., Hirano S., Ikeda M., Sato Y., Iwamura M. (2021). Prognostic impact of AHNAK2 expression in patients treated with radical cystectomy. Cancers.

[28] Chen C., Meng E., Wu S., Lai H., Lu Y., Yang M., Tsao C., Kao C., Chiu Y. (2021). Targeting S1PR1 may result in enhanced migration of cancer cells in bladder carcinoma. Cancers.

[29] Shimura S., Matsumoto K., Shimizu Y., Mochizuki K., Shiono Y., Hirano S., Koguchi D., Ikeda M., Sato Y., Iwamura M. (2021). Serum epiplakin might be a potential serodiagnostic biomarker for bladder cancer. Cancers.

[30] Sugino Y., Sasaki T., Kato M., Masui S., Nishikawa K., Okamoto T., Kajiwara S., Shibahara T., Onishi T., Tanaka S. (2021). Prognostic effect of preoperative psoas muscle Hounsfield Unit at radical cystectomy for bladder cancer. Cancers.

